# Novel Alzheimer’s disease subtypes identified using a data and knowledge driven strategy

**DOI:** 10.1038/s41598-020-57785-2

**Published:** 2020-01-28

**Authors:** Alexis Mitelpunkt, Tal Galili, Tal Kozlovski, Noa Bregman, Netta Shachar, Mira Markus-Kalish, Yoav Benjamini

**Affiliations:** 1 Department of Statistics and Operations Research, Tel Aviv University, Tel Aviv, Israel; 2 Pediatric Neurology Institute, “Dana-Dwek” Children’s Hospital, Tel Aviv Medical Center, Tel Aviv, Israel; 3 Sackler faculty of Medicine, Tel Aviv University, Tel Aviv, Israel; 4 Department of Neurology, Tel Aviv Sourasky Medical Center, Tel Aviv, Israel; 5 The Sagol School for Neurosciences, Tel Aviv University, Tel Aviv, Israel

**Keywords:** Alzheimer's disease, Alzheimer's disease, Information technology, Statistics

## Abstract

The population of adults with Alzheimer’s disease (AD) varies in needs and outcomes. The heterogeneity of current AD diagnostic subgroups impedes the use of data analytics in clinical trial design and translation of findings into improved care. The purpose of this project was to define more clinically-homogeneous groups of AD patients and link clinical characteristics with biological markers. We used an innovative big data analysis strategy, the 3C strategy, that incorporates medical knowledge into the data analysis process. A large set of preprocessed AD Neuroimaging Initiative (ADNI) data was analyzed with 3C. The data analysis yielded 6 new disease subtypes, which differ from the assigned diagnosis types and present different patterns of clinical measures and potential biomarkers. Two of the subtypes, “Anosognosia dementia” and “Insightful dementia”, differentiate between severe participants based on clinical characteristics and biomarkers. The “Uncompensated mild cognitive impairment (MCI)” subtype, demonstrates clinical, demographic and imaging differences from the “Affective MCI” subtype. Differences were also observed between the “Worried Well” and “Healthy” clusters. The use of data-driven analysis yielded sub-phenotypic clinical clusters that go beyond current diagnoses and are associated with biomarkers. Such homogenous sub-groups can potentially form the basis for enhancement of brain medicine research.

## Introduction

Alzheimer’s disease (AD) is a degenerative brain disease and the most common cause of dementia^1^ according to the 2018 Alzheimer’s association report^2^ an estimated 5.7 million Americans of all ages are living with AD in 2018. The percentage of people with AD increases with age: 3% of people age 65–74, 17% of people age 75–84, and 32% of people age 85 and older have AD^3^. Symptoms vary among people with AD, and the differences between typical age-related cognitive changes and early signs of AD can be subtle. The definite diagnosis of AD, requiring histopathological examination, is characterized by the accumulation of β-amyloid (Aβ) plaques and neurofibrillary tangles composed of tau amyloid fibrils associated with brain cell damage and neurodegeneration^4^. In clinical practice, the diagnosis of AD is based on clinical criteria, while laboratory and imaging examinations are used to exclude other diagnoses.

Sub classification of AD has been previously attempted, mostly based on a small set of parameters or on a single modality^5,6^, and in some studies has relied only on previous knowledge. Current diagnostic subgroupings are informative, however, they are quite crude as they are based on rough criteria^7,8^. This may lead astray supervised data mining tools that rely solely on these definitions while trying to predict or associate disease manifestation with clinical and biological markers. Thus, for the search of new insights, it is essential to use unsupervised processes, which do not rely on the current diagnostic subgroupings, Nevertheless, despite numerous attempts to use unsupervised processes as prognostic tools^9^, a specific role for these measures in clinical practice has not been established. A possible explanation for this difficulty is that the pathological findings represent a common advanced stage of a few distinct pathophysiological entities. Those may differ in their clinical manifestations and biomarkers^10–12^. Therefore, an approach that makes use of a large number of clinical and biological markers and finds a way connect these two may offer the desired insight.

Indeed, analyzing medical Big Data, such as the one compiled by the Alzheimer’s Disease Neuroimaging Initiative (ADNI), may promote the understanding of AD mechanisms, prediction of disease courses and, ultimately, assist in identifying potential therapeutic options. However, the analysis of big healthcare data has been fraught with challenges. Associations between biological markers (i.e. imaging, pathology, and genetics) and disease manifestations may be hard to discover. Such associations are especially difficult to find for neurological and psychiatric conditions, as compensatory mechanisms are very common. Still, once discovered, they shed light on interesting pathophysiological processes and may offer directions for developing precise treatments.

Another challenge, which is not unique to medical data, is coping with apparently interesting yet irrelevant discoveries that arise by mere chance, due to the extensive search conducted. Irrelevant results can be avoided by splitting the data into training and validation samples, if the data is vertical (long) with many more subjects than features. If the data has few subjects per feature, or even less than one (horizontal, or wide data) which is common in medical big data, the challenge is greater.

In this study, we set out to identify diagnostic subtypes and their unique signatures^13^ among the AD population using the 3C strategy^14^ for medical Big Data analysis. This strategy combines supervised and unsupervised methods and relies very partially on current diagnostic subgroups. It was developed as part of the Human Brain Project^15^ using the Alzheimer’s Disease Neuroimaging Initiative (ADNI) data.

## Results

### Clusters identified in the ADNI database

The 3C methodology progresses in three major steps, where the results of earlier steps serve the following ones. In the Categorize step, the 144 clinical characteristics were screened for association with the assigned diagnosis, and the leading 12 as indicated by VSURF^16^ were selected for unsupervised clustering. Those included: Clinical Dementia Ratings scales of memory, global score, sum of boxes, judgement, Mini Mental Status Examination (MMSE) total score, sum of memory scores, Functional Activities Questionnaire total score, Everyday Cognition study partner’s and participant’s assessment of memory and participant’s overall score, Functional Activities Questionnaire, The Alzheimer’s Disease Assessment Scale overall score and delayed recall/memory score. The original ADNI data consisted of 5 diagnosis groups: AD (Alzheimer’s disease, N = 110); LMCI (Late Mild Cognitive Impairment, N = 133); EMCI (Early Mild Cognitive Impairment, N = 148); SMC (Significant Memory Concern, N = 94); and CN (Cognitively Normal, N = 173).

The Cluster step used the 12 selected clinical measurements (CM) to perform unsupervised clustering into 6 clusters: “Healthy” (cluster 1, N = 121), “Affective Mild Cognitive Impairment (MCI)” (cluster 2, N = 101) “Anosognosia dementia” (cluster 3, N = 54), “Worried Well” (cluster 4, N = 148), “Uncompensated MCI” (cluster 5, N = 135), “Insightful dementia” (cluster 6, N = 98). The diagnosis and cluster assignments are presented in Table 1.

**Table 1:** Number of participants in Each Cluster by Original Diagnostic Group.

Cluster	AD	LMCI	EMCI	SMC	CN	Final Cluster Sum
**Healthy (1)**	0	0	1	25	95	**121**
**Affective MCI (2)**	1	30	70	0	0	**101**
**Anosognosia dementia (3)**	54	0	0	0	0	**54**
**Worried well (4)**	0	0	1	69	78	**148**
**Uncompensated MCI (5)**	2	65	69	0	0	**136**
**Insightful dementia (6)**	53	38	7	0	0	**98**
Original Diagnosis Sum	**110**	**133**	**148**	**94**	**173**	**658**

AD: Alzheimer’s disease; LMCI: Late Mild Cognitive Impairment; EMCI: Early Mild Cognitive Impairment; SMC: Significant Memory Concern; CN: Cognitively Normal.

The Classify step used the clusters to discover potential biomarkers (PB) related to each cluster and perform pairwise comparisons between the clusters in order to identify the significant set of PB for each cluster.

Six clusters were identified. As can be seen in Table 1, the partition of the participants into these clusters differed from their original diagnosis (Dx); only Anosognosia dementia included exclusively participants diagnosed with AD. However, only 49% of all participants diagnosed with AD were included in Anosognosia dementia, and another 48% were in Insightful dementia.

### Demographic characteristics by cluster

Demographic differences between the clusters were small; average age at diagnosis ranged from 69.9 to 74.1 across the 6 clusters with statistically significant differences (p < 0.001, Kruskal-Wallis) and education differed by 1.6; schooling year (p = 0.003 Kruskal-Wallis). The proportion of females ranged from 38–56% and the proportion of participants with Hispanic ethnicity ranged from 2.2–7.4%, although both differences were not statistically significant. (see Table 2 for details).

**Table 2:** Characteristics of clusters population.

Cluster/Variable		Total	1	2	3	4	5	6	p-value
Participants		658	121	101	54	148	135	98	
		Mean (SD)							
Age		72.51 (7.1)	73.0 (5.7)	69.9 (6.5)	74.6 (8.8)	72.8 (6.4)	71.7 (7.8)	74.1 (7.4)	**<0.001**
Education		16.4 (2.6)	16.8 (2.6)	16.8 (2.5)	15.2 (2.4)	16.5 (2.6)	16.2 (2.6)	16.3 (2.8)	**0.003**
		n (%)							
Gender	Male (%)	341 (51.9)	53 (43.8)	55 (54.5)	32 (59.3)	70 (47.3)	71 (52.6)	60 (61.2)	0.097
Ethnicity	Hispanic	25 (3.8)	8 (6.6)	2 (2.0)	4 (7.4)	5 (3.4)	3 (2.2)	3 (3.1)	0.081
	Not Hispanic	628 (95.6)	110 (90.9)	99 (98.0)	50 (92.6)	143 (96.6)	132 (97.8)	94 (95.9)	
	Unknown	4 (0.6)	3 (2.5)	0 (0.0)	0 (0.0)	0 (0.0)	0 (0.0)	1 (1.0)	
APOE4	0	280 (53.8)	64 (68.8)	58 (58.6)	13 (29.5)	56 (71.8)	63 (50.0)	26 (32.5)	**<0.001**
	1	187 (36.0)	26 (28.0)	34 (34.3)	23 (52.3)	19 (24.4)	46 (36.5)	39 (48.8)	
	2	53 (10.2)	3 (3.2)	7 (7.1)	8 (18.2)	3 (3.8)	17 (13.5)	15 (18.8)	

### Clinical characteristics of the new clusters

#### Clusters offer a more refined clinical distinction than diagnosis subgroups

**Figure 1:**
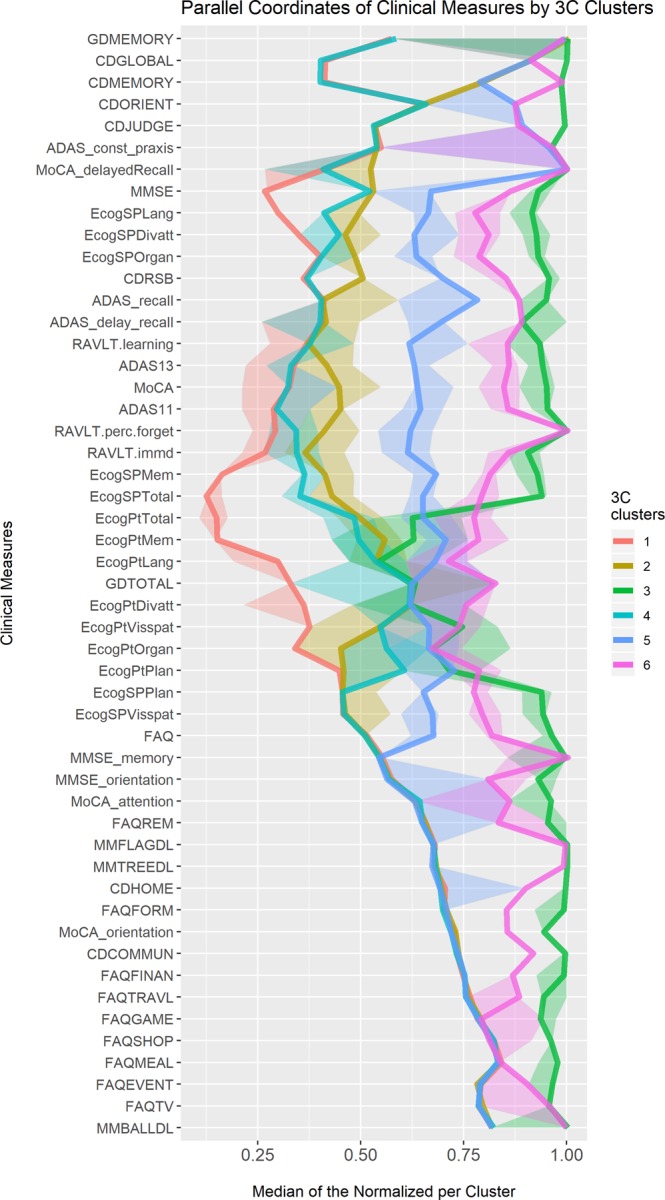
Parallel coordinates plots of clinical measures for new clusters. A parallel coordinates plot of subtypes across CM. The variables identified on the Y-axis include the 12 CM that were selected for building the clusters using Random Forest. The additional 28 were selected by retaining a significant difference in at least one comparison between the subtypes after adjusting for multiple comparisons per variable. For each of the presented CM, the observations were transformed to their empirical quantiles where 0 represents the healthy state of each variable and 1 represents the most severe state. The median value for each of the subtypes is presented in the X-axis, connected across the different CM. A 95% confidence interval for each of the median lines, added as shaded ribbons, are based on quantiles of the binomial distribution. The CM variables were ordered so that CM with similar pattern will be closer to one another. CM - clinical measures. Interactive version available Supplementary Fig. S1.

Figure 1 is a parallel coordinates plot, in which the values in each cluster of the 12 CM used for the clustering, with 28 other measurements that were also found to differ between the six clusters in a statistically significant way (adjusted for their selection) are presented.

Each cluster is displayed by a broken line, and it is evident that the new clusters are easily distinguishable. Moreover, the confidence intervals (CIs), represented by the shade around each line, are also well-separated, further supporting a robust inter-cluster differentiation of the new clusters. In contrast, a similar plot based on the original diagnosis groups shows 4 out of the 5 to have considerable overlap (see interactive versions supplementary Fig. S1 and supplementary Fig. S2).

#### Clinical features of individual clusters

Below are selected clinical features of the clusters, presented in order of disease severity.

Anosognosia Dementia subtype (Cluster 3). For most CMs, the values of this cluster are at close to the maximum severity. CMs with lower values (i.e., less severe), belong to the Everyday Cognition questionnaire self-reported by the participant (ECogPT). This cluster was defined as the Anosognosia Dementia because of the particular combination of severe cognitive impairment with lack of self-awareness. Disturbances of awareness in AD can have a far-reaching impact on both diagnosis and treatment. They might affect readiness to seek diagnosis, predict disease progression^17^ and determine treatment compliance^18,19^. Anosognosia Dementia includes only participants that were defined by ADNI as AD, i.e. participants with diagnosis of probable AD.

Insightful Dementia subtype (Cluster 6). The curve plotted for the cluster is easily distinguishable from that of Anosognosia Dementia, as the values for CMs are not quite as high as in Anosognosia Dementia. The highest values, similar to those of Anosognosia Dementia, are for memory-related CMs, specifically memory domain values within general cognitive assessments: MMSE, Montreal Cognitive Assessment (MoCA) test and Rey Auditory Verbal Learning Test (RAVLT). The major difference between Anosognosia Dementia and Insightful dementia was the participants’ own appreciation of their difficulties, meaning lower levels of insight and awareness to their disease. These phenomena might reflect a different mechanism and/or disease spread in terms of the brain areas involved. A longitudinal follow up might answer the important question of whether the two populations of participants have different courses with respect to disease progression. Insightful dementia includes mostly participants with diagnosis of AD dementia, and fewer with LMCI (54.2% and 38.7% of this cluster population, respectively). Only 7.1% are EMCI.

Uncompensated MCI subtype (Cluster 5). Most of the CM values are in the middle range of functional level. The highest values (most severe) are in CMs that represent short- and intermediate- term memory especially language-related memory tests. In Fig. 1, the variables with higher values are delayed word recall, word recall, delayed recall from the MoCA and constructional praxis. Uncompensated MCI includes mostly participants with diagnosis of MCI, with very similar proportions of LMCI and EMCI (47.8% and 50.7% respectively). The remaining 1.5% are AD.

Affective MCI subtype (Cluster 2). Significant demographic differences can be observed in Table 2. Two CMs have very high (transformed) values; two of these variables are the Geriatric Depression Scale (GDS): the summed score (GDTOTAL) and and the memory-related questions (GDMemory). Two additional variables of Clinical Dementia Rating CDGLOBAL and CDMEMORY which are higher than the values in clusters 1 and 4. Affective MCI contains mostly participants with the original diagnosis of EMCI, and a much smaller proportion of LMCI (69.3% and 29.7% respectively). Only one participant with a diagnosis of AD was included in this cluster.

Worried well subtype (Cluster 4). Consists almost exclusively of participants without any diagnosis in the spectrum of cognitive impairment. CMs that represent the participant’s overall, current memory and language self-assessment of Everyday Cognition (ECog) as compared to ten years earlier are higher in this cluster than in Healthy. Slightly lower are the similar respective variables of the assessment by the participant’s partner, which are higher in Uncompensated MCI than in the Healthy cluster.

Healthy (Cluster 1). Its features are at the bottom (best function) of the parallel coordinated plot, hence, will be referred to as the Cognitively-Normal (CN) subtype.

### Potential biomarkers for the new clusters

**Figure 2:**
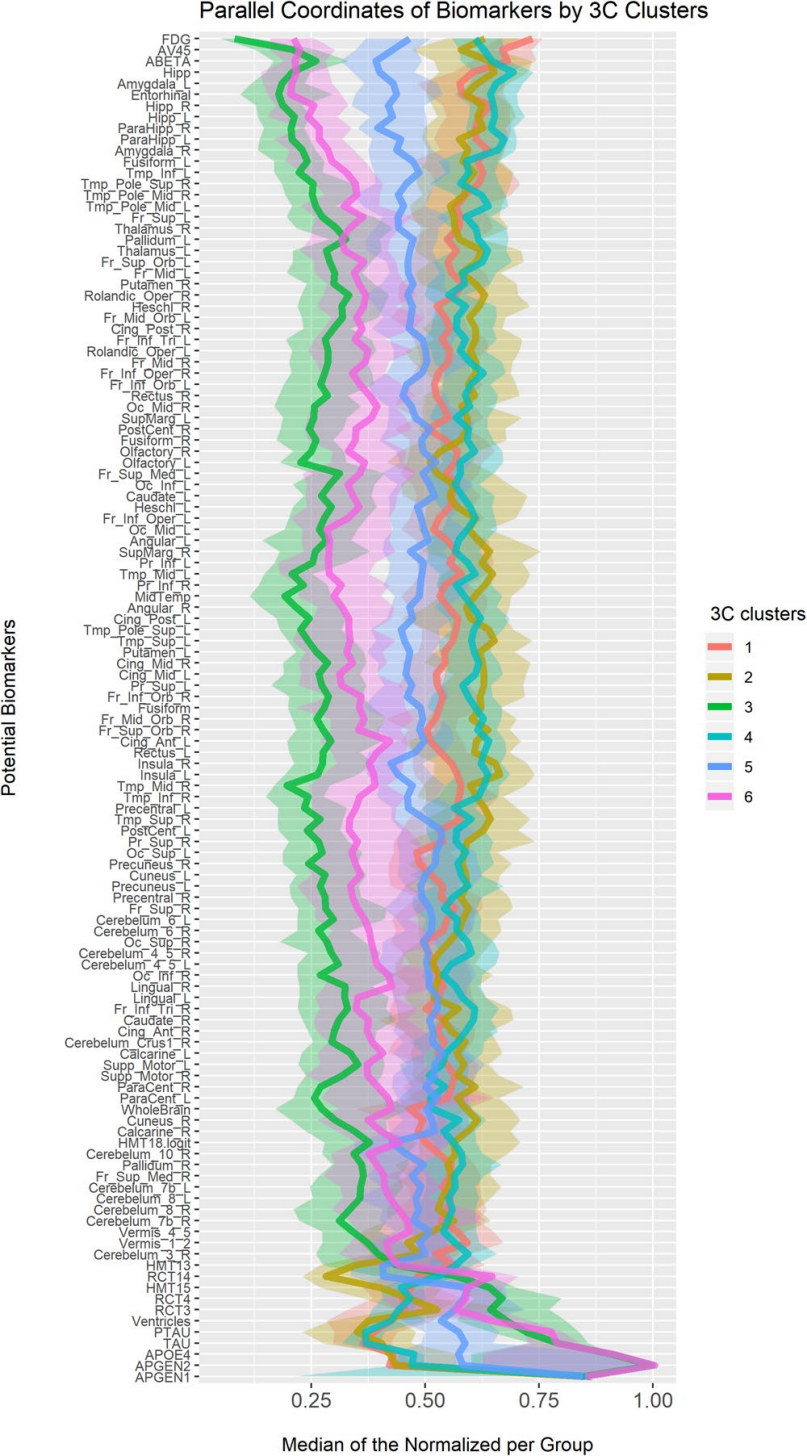
Parallel coordinates plots of potential biomarkers for new clusters. A parallel coordinates plot of subtypes across pBM. The variables identified on the Y-axis include the pBM that were selected by retaining a significant difference in at least one comparison between the subtypes following adjustments for multiple comparisons. For each of the presented pBM, the observations were transformed to their empirical quantiles where 0 represents the healthy state of each variable and 1 represents the most severe state. The median value for each of the subtypes is presented in the X-axis, connected across the different pBM. A 95% confidence interval for each of the median lines, added as shaded ribbons, are based on quantiles of the binomial distribution. The pBM variables were ordered so that pBM with similar pattern will be closer to one another. pBM - potential biomarker. Interactive version available Supplementary Fig. S3.

The preliminary collection of pBM encompassed 170 variables. Screening for those that were significantly associated with any of the clusters, yielded mostly volume measurements of brain regions (attained through imaging). Other pBMs significantly associated with clusters included blood tests and measures of other imaging modalities (i.e. fluorodeoxyglucose positron emission tomography [FDG-PET], magnetic resonance imaging [MRI]) (The full list of biomarkers selected for the analysis is provided in Supplemental Fig. 3). An analysis presented in the methods section was performed, this time with the selected pBM and using the newly-defined clusters as target, and results are presented in Fig. 2.

#### Clusters offer a more refined distinction by biomarkers

As with the analysis by CM, the new clusters presented in Fig. 2 show less overlap between plots of clusters than the overlap in a similar display making use of the original ADNI diagnoses (interactive versions supplementary Fig. S3 and supplementary Fig. S4). This lends further credibility to the new clustering, seeing as the clusters were constructed based on clinical features, and yet provide a better differentiation between clusters by biomarkers. In Anosognosia Dementia, the areas of atrophy are dispersed throughout the whole brain compared to volume calculated for other clusters. Insightful dementia is easily distinguishable from Anosognosia Dementia in this analysis, and even more so from Uncompensated MCI, which presents an intermediate pattern of atrophy between the severe phenotype of Anosognosia Dementia and Insightful dementia, and the milder one of clusters Healthy, Affective MCI and Worried Well. For Insightful dementia, the clinical distinctions are supported by the biomarkers that are preserved compared to Anosognosia Dementia. These areas include Right Cuneus, Right Occipital, Calcarine, Cerebellum, Frontal, Lingual, Pallidum, Paracentral and Vermis.

In Uncompensated MCI, the brain regions with significantly smaller volumes include: Left hemisphere Cingulum, Fronto orbital, Fusiform and Putamen. Bilateral atrophy was found in the Amygdala, Entorhinal, Hippocampus, ParaHippocampal, and Rectus regions. There is a difference between Affective MCI and Insightful dementia regarding total Tau (tTau) and hyperphosphorylated Tau (pTau), which reflect the degree of active neurodegeneration^20^.

#### Biomarkers for individual clusters

**Figure 3:**
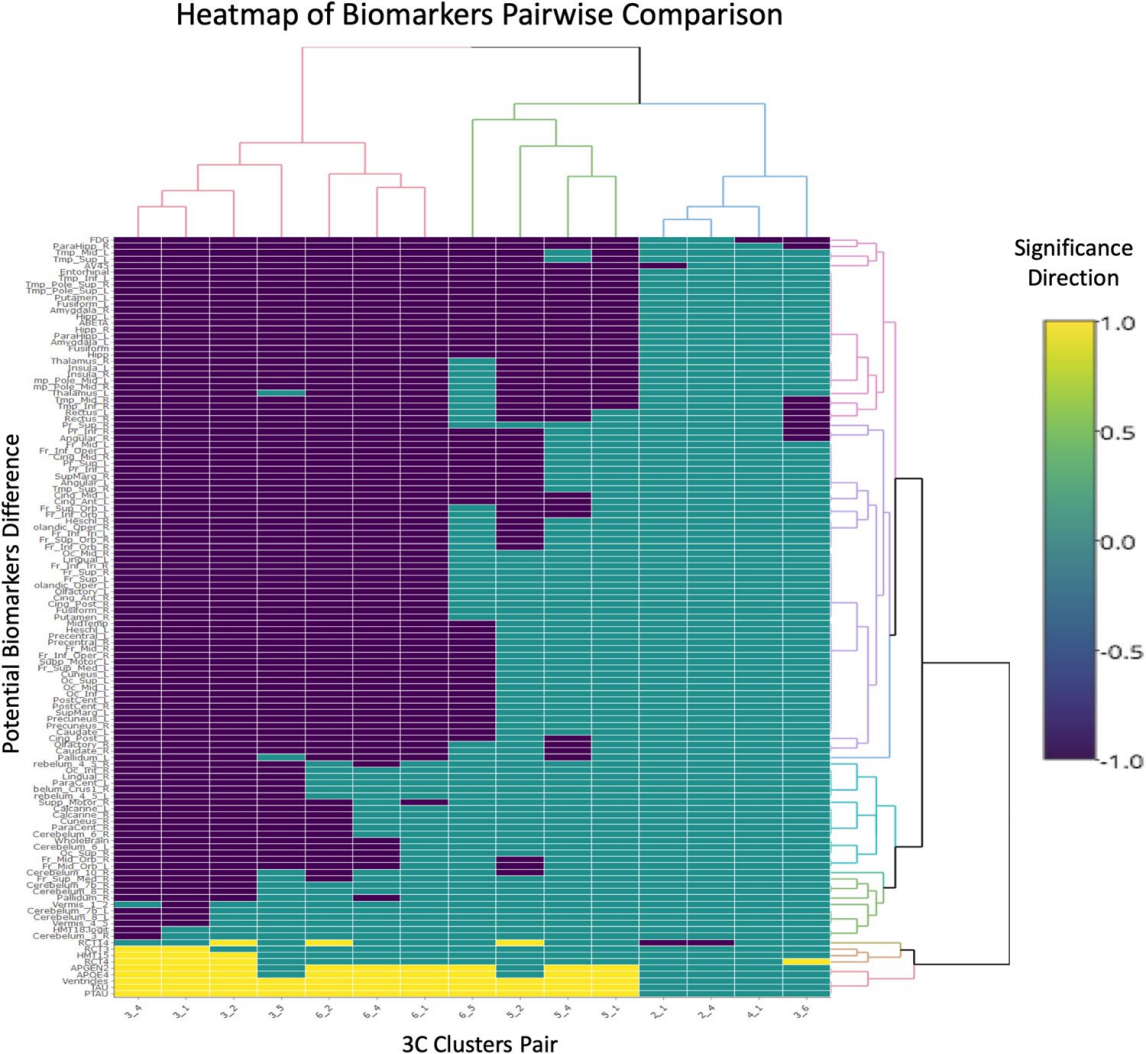
Heatmap of potential markers pairwise comparisons across new clusters. Cluster heatmap of pBM for which there was a significant difference between the subtypes, found using the Kruskal-Wallis test with an FDR control using the BH method. Each row represents one pBM and each column represents a comparison of two of the six subtypes. There are 15 such comparisons/columns. Each cell is colored in either turquoise, purple or yellow. Turquoise cells indicate non-significance comparisons of pBMs. For column x, a cell at row y colored in yellow indicates that x has a significantly larger center (mean rank) than y, while a purple color would indicate that y is significantly larger than x (using Wilcoxon test). The dendrograms of the heatmap helps in sorting the rows and columns so to highlight interesting patterns. The pBM with significant difference between subtypes 3 (anosognosia dementia) and 6 (insightful dementia) marked by black arrows. The significant differences between subtypes 5 (Uncompensated MCI) and 2 (Affective MCI) in marked by the black box with white asterisk. pBM - potential Biomarkers, MCI - minimal cognitive impairment. Interactive version available Supplementary Fig. S5.

The heat map provided in Fig. 3 is a visual representation of biomarkers that have the potential to differentiate between the clusters (interactive version Supplementary Fig. S5) For each of these pBM, a pairwise comparison was conducted, with corrections for multiple comparisons again using the Benjamini-Hochberg procedure (BH)procedure to control False Discovery Rate (FDR)^21^. Finally, hierarchical clustering algorithms were used to generate the map.

**Figure 4:**
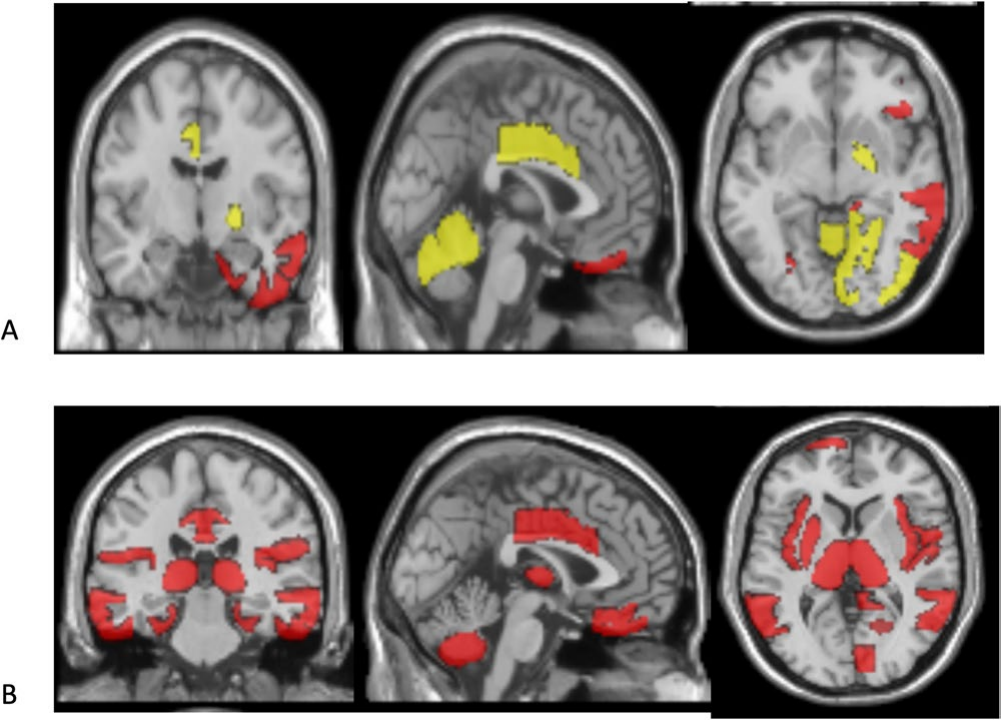
Difference of brain regions volumes between clusters. Differences in Brain region volumes between subtypes. (**A**) The regions with significant difference between subtypes anosognosia dementia and insightful dementia compared to all other subtype (as reference) are marked by yellow areas. The red regions indicate additional regions with significant difference between subtype anosognosia dementia and all other subtypes (difference did not reach significant difference compared to insightful dementia subtype). (**B**) Regions with significant differences between subtypes uncompensated MCI and Affective MCI. All pairwise comparison where corrected for multiple comparisons. Figure created using mricron software (http://people.cas.sc.edu/rorden/mricron/index.html).

The bulk of the purple areas of the map represent brain regions in which atrophy was observed in participants from Anosognosia dementia and Insightful dementia, but not the other clusters. The top right corner of the map represents areas in the brain that were found to have significantly smaller volumes in brains of participants from Anosognosia Dementia compared to participants from Insightful dementia. The top center region of the map represents areas in the brain that showed a significant level of atrophy in participants from Uncompensated MCI compared to those in clusters Healthy, Affective MCI and Worried Well. For Affective MCI, the biomarker showing significant difference from all other subtypes is the creatine kinase plasma level. As for Healthy, some pBM were selected for this subtype, but those would be interpreted as the “healthy” reference when comparing to other clusters. Uncompensated MCI differs from Healthy in the level of FDG_PET might be early signs of neurodegeneration before atrophy occurs. The differences are also presented in Fig. 4.

## Discussion

This study illustrates the heterogeneity within the population of adults with AD and the potential power of the 3C methodology to uncover subgroups. Six new distinct clusters were identified that are easily distinguishable from one another, with a better resolution of separation by clinical features and biomarkers than provided by the original diagnoses. The new clusters shed light on functional diversities in cognitive domains. Their correlations with the biomarkers generate subtype signatures, which in turn can assist in improving patient tailored treatment and in focusing research on distinct disease mechanisms. We labeled the new clusters relying on known clinical entities and described them using additional sub-phenotyping and biomarker descriptions.

Three important insights stem from our analysis: first, participants with the original diagnosis of AD were divided between two clusters. These clusters differ in self-assessment of the participant as compared to their partners. The discrepancy appearing in Anosognosia Dementia can be interpreted as a representation of the decreased ability to recognize their disorder, a symptom which is common in the clinical presentation of AD^22,23^. The Insightful Dementia cluster participants presented largely better clinical features, with differentiating CMs being mainly related to measurements of general function. They also exhibited preserved brain regions including the right temporal and parietal lobes and bilateral orbitofrontal cortex (rectus gyri), right cuneus, right occipital, calcarine, cerebelum, frontal, lingual, pallidum, paracentral and vermis. These brain regions have been reported in relation to specific AD subtypes^10,12,24–31^. The preserved frontal areas could potentially explain the differences in insight and executive function resulting in a better functional state.

Second, the Uncompensated MCI subtype received the term because several CMs across cognitive domains were decreased rather than the dominance of memory decline expected in AD. Within the memory domain the worst results were in language-related memory tests. Participnats in the Uncompensated MCI cluster presented an uncommon pattern of brain atrophy; the hippocampal area was relatively preserved in observed median volume compared to the Anosognosia Dementia and Insightful Dementia subtypes. Left hemisphere dominance in the atrophied regions could reflect the relation to language and semantic brain areas^32–35^. The clinical finding is supported by the Left hemisphere Cingulum, Frontal inferior operculum, Fusiform^36^ Superior Parietal and Putamen had significantly more reduced volume in Uncompensated MCI compared to Affective MCI. Bilateral atrophy was found in the Amygdala, Entorhinal, Hippocampus, ParaHippocampal, Frontal_orbital, Temporal and Rectus regions. It is interesting that participants assigned to the Uncompensated MCI cluster had more constructional apraxia which is considered to be right hemisphere-parietal mediated. It is consistent with the greater atrophy observed in this cluster in the right Parietal lobe in the pBM. Right Hemisphere – Para hippocampus area, right Parietal which could explain the difference in praxis. Differences in bilateral rectus (orbitofrontal) volumes could be related to decreased awareness to disease condition and worse judgment. This relation between functional and biological patterns is a good example of the importance of disease subtype signatures.

Affective MCI cluster was given this term as this cluster of younger and more educated patients (as seen in Table 2) had original diagnoses of early and late MCI. The most striking functional difference from the Healthy cluster was seen in the GDS variables GDTOTAL and GDMEMORY. Significant differences in values were not found in other memory tests. This cluster was also differentiated from all other subtypes by a high value of the biomarker level of creatine kinase, which has been reported previously to be related to dementia^37,38^. Relation between AD and depression has been reported^23^. The younger and more educated generally have more awareness of their disease and are therefore at risk of developing depression in the early stages of cognitive impairment. Whether treating the depression aspects would yield better functional results in other domains is yet to be studied.

A possible explanation for the differences between the better performing subtypes in both dementia and MCI could be related to the lesser involvement of frontal brain regions.

The depression might be the cause for cognitive decline, but it might as well be a result of better awareness, insight and judgment abilities in people encountering memory impairment.

Younger and more educated people have greater chances of having more cognitive reserve^39,40^, and this could translate to improved overall function thanks to better adaptive compensatory mechanisms.

The ADNI separation of EMCI and LMCI is not in line with the results of our study, as is shown in Table 1: LMCI: 22.5%, 48.9% and 28.6% in clusters Uncompensated MCI compared to Affective MCI and Insightful dementia respectively. EMCI: 47.3%, 46.6% and 4.7% in clusters Uncompensated MCI compared to Affective MCI and Insightful dementia respectively. None were designated to Anosognosia Dementia, and about a quarter of LMCI in Insightful dementia – probably reflecting those with the worse cognitive impairment that are still not classified as dementia (meaning – good daily function). Thus, with the 3-C method the original ADNI classes are dismantled and redistributed, yielding new and more clinically homogeneous sub-types, with a higher degree of correlation to specific biomarkers.

The results should be considered with the caution and they should be validated in future studies. Moreover, they are still based on clinical phenotypic subgroups and not solely on biological endophenotypes. Nevertheless, the subtypes found in this study may support improvement in clinical and translational research. Scenarios in which these subtypes may be valuable include the following: 1. Disturbances of awareness in AD can have a far-reaching impact on both diagnosis and treatment. They might affect readiness to seek diagnosis, predict disease progression^17^, and determine treatment compliance or the ability to sign informed consent. Lack of awareness of deficits in AD is associated with impairment in daily functioning^41^, with behavioral disturbances^42^, as well as with overall severity of cognitive impairment^22,43,44^. 2. Assessing the relation of frontal atrophy or other biomarkers of involvement to the level of anosognosia. Early awareness of increased risk of developing anosognosia has clinical value in assisting patients and families to properly prepare (legally, emotionally, financially, socially etc.) to a state of anosognosia. 3. Focused research and clinical trials could benefit from selection of sub-groups of patients with higher risk of developing anosognosia based of imaging markers. 4. Biomarkers and imaging markers suggestive of Affective MCI, upon validation in larger, targeted, biomarker-driven research, have translational importance to direct physician’s attention to affective disorder symptoms, needs and treatment. These are in many cases under diagnosed and under treated^45^.

One way that the 3C strategy addresses the crudeness of currently assigned diagnoses by increasing the number of clinical variables used to determine the diagnosis and broadening their scope. The ADNI study makes use of six variables whereas, the 3-C strategy allowed the use of hundreds of available variables. The use of the assigned diagnosis at the feature screening stage, ensures that the available clinical knowledge is not ignored. Creating subtypes of the disease based on data-driven selection of clinical measurements is an additional ingredient of the strategy that allows for formation of more homogenous groups.

The strategy supports CM that represent symptoms and signs that have strong evidence of direct relation to the disease. In the case of AD, the existing biomarkers are not specific enough yet to support a concrete outcome and further studies establishing these relations are needed.

Each subtype is linked to a clinical measurements representation and a set of potential biomarkers. Subtypes are based on the clinical measures available to the physician and coupled to a set of biomarkers.

The entire analysis process is governed by statistical means such as the control of the FDR in screening testing and prediction. These tools were assessed and modified using a simulation study^46^ to find the most appropriate process minimizing the chances of finding irrelevant solution – a risk inherent in the analysis of Big Data.

In the future, when large biomarker-driven datasets will emerge as presented by Espay *et al*.^47^ the 3-C strategy will be useful for an iterative process of knowledge discovery. Moreover, the process provides assertion that the biomarker discoveries are reliably related to clinical implication. In the meantime, 3-C can be used as a bridging strategy between symptoms-driven and biomarker-driven approaches, gaining more knowledge from existing cohorts.

The translational value of these subtypes can take different directions: clinical trials could have more precise treatment assignments according to subtype; subtype characteristics can drive research aimed at understanding pathological mechanisms; and physicians can assess the subtype which their patient is most likely to resemble and illuminate the predicted disease trajectory and suitable treatment.

The insights mentioned are encouraging, yet some limitations exist. Even though the derived clusters show more distinct groups in the biomarkers than the original diagnoses (even the CN diagnosed group crossed the median percentile and “mixed” with the other clusters in a parallel coordinate plot), we did not find complete separation of the clusters, as demonstrated by the standard deviation information added to the parallel plots. The proposed biomarkers as well as the new subtypes related to them require further confirmation in additional targeted studies. Once confirmed, the clinical and biomarker application to clinical trials can become useful. The use of baseline cross-sectional data serves the purpose of creating a model assisting at the diagnostic stage but lacks the progression aspect. Use of the ADNI data has an inherent bias as it is not a random sample of the general population. A platform such as the Medical Informatics Platform (MIP) of the human brain project (HBP) that facilitates patient-privacy-preserving access to hospital data which is both larger as well as contained an unselected set of subjects, has the potential to provide a sample which is more representative of the general AD population.

The assignment of a diagnosis based on clinical assessment even for dimensionality reduction has a limitation not being a biologically defined cause^47^. Diagnosing a neurological disease presents even greater limitation, as histological information is rare and the interaction of a person’s history, compensatory mechanisms and variety of non-specific symptoms may obscure diagnosis at early stages. Future discoveries of well-established biomarkers and their clinical outcomes will gradually reduce the ambiguity. The described 3-C strategy is one method that could support such a discovery process.

Further research should be done to evaluate the medical and technical challenges. The number of clusters will be based on medical knowledge, however, improved statistical methods to suggest possibilities for appropriate numbers of clusters and ways to combine specific domains of knowledge in real life problems should be developed. Multi categorical prediction models can assist in facing healthcare challenges. Medical prediction models on conversion rates from CN to MCI or AD, as soon as enough data accumulates on the participants, may assist in verification and improvement of the models. These findings need to be validated by replication in another set of subjects, applying the methodology on a different disease as well as other data sets of dementia studies.

New MCI and dementia subtypes were identified in a data-driven and medical knowledge incorporated process. The possibility to redefine diagnostic subtypes and finding disease signatures are promising directions bringing future medicine closer. The 3-C strategy for big data analysis in medical informatics addresses identified challenges in the process, and the results of the analysis show further translational benefits by focusing the clinician and researchers on the sets of important clinical measurements and biomarkers.

## Methods

This informatics driven study was conducted using the data from ADNI.

### Alzheimer’s disease neuroimaging initiative (ADNI)

Data was extracted from the ADNIMERGE R package, downloaded on August 26, 2014 (see www.adni-info.org available for download at the ADNI website). Up to the cutoff date of August 26, 2014 1736 adults, ages 55 to 90, have been recruited to participate in the study. The study reported in this paper was conducted on data collected at baseline visit of the ADNI-2 and ADNI-Go parts of the study including 917 participants. The decision to focus on this group was based on the fact that it has the widest possible range of clinical and neuropsychological data. 197 features (variables) were identified as relevant.

**Figure 5:**
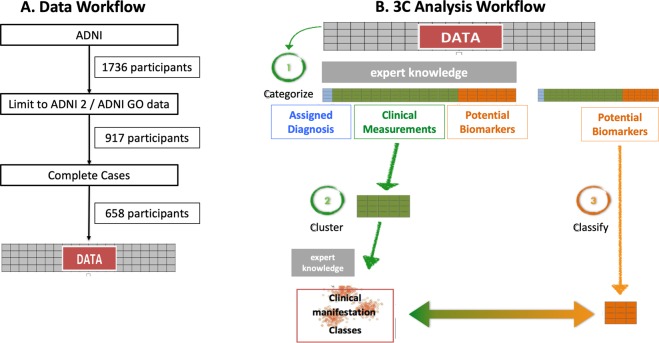
Data and 3C analysis workflows. (**A**) Workflow of dataset creation, ADNI 2/ADNI GO were selected for their extended clinical data variables. The final dataset consisted of complete cases only. (**B**) 3C analysis methodology workflow. A schematic presentation of the three steps. 1 Categorization of data into assigned diagnoses, clinical measures and potential biomarkers. 2 Clustering based on the clinical measures. 3.Classifications of biomarkers to the created clinical clusters. ADNI - Alzheimer’s Disease Neuroimaging Initiative.

Initial preprocessing of the data revealed that missing values were highly informative of disease diagnosis: for example, some measurements were less available for healthy subjects, while ill subjects often had most measurements available. We therefore kept variables and subjects so that no values were missing. This step led to a reduction in the number of variables to 191 and the number of observations from 917 to 658, data workflow presented in Fig. 5.

The data was further pre-processed in various ways. Variables with correlation >0.99 or for discrete ones with Gini impurity <0.05 were removed. New variables were created by summing scores per cognitive domain in cognitive exams, and monotone non-linear transformations of the variables were used to improve symmetry, linearity and homogeneity of variances following the methodology described in Shachar *et al*.^48^.

### The 3C data analysis strategy

A three-step data analysis strategy described in Galili, Mitelpunkt *et al*.^49^ was used to categorize, cluster and classify (3C) the data. The 3C strategy has been newly developed and tested with the ADNI dataset. It is designed to integrate both supervised and unsupervised methods in order not to rely solely on assigned current diagnosis, and at the same time overcome the challenges faced with analyzing big data. It comprises three stages. (i) Based on current knowledge of medical expertise, variables from the ADNI (features) are categorized into either: Assigned Diagnosis (DX); clinical measurements (CM) representing the manifestation of the disease; or potential biomarkers (pBM) that have been collected from the patient but their relationship to the disease manifestation is not yet fully asserted. (ii) The CM are screened for relevance using the assigned diagnoses variable as guides. The selected CM are clustered using unsupervised data algorithms to identify homogenous clusters of disease manifestation. The number of clusters is determined by combining statistical criteria with medical interpretation. (iii) These clusters can be regarded as newly defined subtypes of the disease, which are in turn classified by the biomarkers to the newly defined disease subtypes. This relationship represents a “disease subtype signature”, Fig. 5 illustrates the Analysis workflow.

#### Categorize

First, the ADNI data was categorized by a medical expert (A.M.), into three categories:

1. **Assigned diagnosis (DX):** Based on the ADNI2 procedure manual(ref), participants were assigned one of five diagnoses: AD (Alzheimer’s disease); LMCI (Late Mild Cognitive Impairment); EMCI (Early Mild Cognitive Impairment); SMC (Significant Memory Concern); CN (Cognitively Normal). The ADNI2_procedure_manual^50^, makes use of thresholds on six inclusion variables: (1) Mini-Mental State Examination, (2) Cognitive Dementia Rating sum of boxes, (3) patient and partner assessment of memory and overall function, (4) medical overall estimation, (5)stability of medications and (6) Geriatric Depression Scale.
2. **Clinical measurements (CM):** A total of 144 variables were categorized as Clinical measurements reflecting the patient’s condition and functionality. In the case of cognitive impairment, they encompassed scores of different Neuropsychological tests’ ratings and standard questionnaires (of participant or study partner (SP) reports), which are part of the clinical assessment and diagnostic process. Other CMs included differential diagnoses and co-morbidities such as depression or other psychiatric disorders and demographic data.
3. **Potential biomarkers (pBM):** A total of 170 variables were categorized as pBM by a neurologist (A.M.), including biochemical measures in blood and in cerebrospinal fluid (CSF), genetic status and susceptibility, PET (FDG and Amyloid) findings and MRI findings. Specifically, MRI-extracted features included both volumetric measures of brain regions calculated by ADNI researchers, and additional higher resolution volumetric variables came from MRI data that were processed to extract information about the volume of brain regions in individual patient brains using Statistical Parametric Mapping (SPM). These variables do not describe the functional status of the participant nor were there any guidelines in the literature associating them to a specific condition. Yet, they function as biomarkers that could potentially be related to the disease mechanisms or state.

#### Cluster

Next, the set of clinical measurements was clustered using k-medoids unsupervised clustering with the Manhattan distance metrics, preceded by a screening stage for relevance in order to avoid error-ridden clusters. In order to screen for their relevance, the explanatory power of each variable with the assigned diagnoses as targets was assessed by using variable importance in random forests^51^ - while considering the background contributions of the entire set of CM features. 12 CM out of the 144 screened are detailed in the Results section. The screening step enables to leverage the knowledge used for devising DX, while expanding beyond the existing set of diagnosis codes and not addressing them as the ultimate truth. Yet, the variables screened relate to the disease instead of ignoring it completely, this demonstrates an additional way to incorporate the medical knowledge into the data analysis process. In order to determine the appropriate number of clusters we again used a combination of medical knowledge and statistical indications. We used the GapStatistics^52^ as a first step for choosing several potential number of clusters. The first drop was after three clusters, the second after 8, indicating 3–8 as the potential number of clusters. From the literature^5,6,10,12,24,25,53–55^ and knowledge about dementia, several sub-classes of patients are known within the clinical spectrum between Normal and AD. A neurology expert (A.M.) inspected each potential cluster formation using parallel coordinates visualizing the 40 variables as in Fig. 1, and a scatter plot of the first two principal components.

#### Classify

Finally, these clusters were used as a new set of clinical subtypes. The pBM were now screened to identify those markers that differed statistically significantly using Kruskal-Wallis test between these newly defined subtypes. For the biomarkers that showed some differences, all pairwise differences between the clusters were tested, using the Wilcoxon Rank Sum test. The BH procedure^21^ was used to set the FDR at 0.05. It is important to emphasize that the pBM were not used for the construction of clusters only CM, so the classification was not using the same variables as the clustering step.

### Algorithms and software

The algorithms for clustering and feature selection were chosen based on a simulation study that compared several different algorithms^46^. The screening step uses variable importance of each variable in the random forests (via the VSURF R package^56^) in order to address the challenges of dimensionality^57^ encountered when trying to cluster high dimensional data that includes irrelevant variables. The chosen number was based on the software defaults, using two of the three steps in VSURF^16^ (“thresholding step” and “intepretation step”, described in their original paper, nfor.thres = 50 and nfor.interp = 25). Clustering was done using k-medoids with Manhattan distance. We used the GapStatistics^52^ combined with prior clinical understanding, to choose to use 6 clusters. The non-parametric Kruskal-Wallis test was used to identify pBM that showed differences across clusters, while controlling the FDR at 0.05. For the identified pBM, pairs of clusters were tested for differences, using the two-sample Wilcoxon Rank-sum tests. Again, FDR adjustment was made, but now at the lower level of 0.05*51/170 because of the screening that was already made, per the algorithm of Benjamini and Bogomolov^58^. FDR adjustments were calculated using the BH procedure p-value adjustment procedure in R (p-adjust). Further details on the methods used throughout 3C are available^59^.

All images passed through a visual quality control step before further analysis. All subjects’ grey matter volumes were estimated using SPM12^60^, an open source software package written in Matlab (Mathworks, Natick, MA). Image pre-processing consisted of a number of steps including adjustment to gender and age, unified segmentation, deformation with the Dartel algorithm and modulation followed by smoothing with an isotropic Gaussian kernel with FWHM = 8 mm since the voxel size of ADNI images from 1.5 T was 1 × 1 × 1.2 mm from 3 T was 1 × 1 × 1.2 mm as well and the 8 × 8 × 8 mm cornel to smooth the data was applicable in this situation.

Details about the construction of the figures are presented in their legends, and pointers to their interactive versions online are given there.

### Ethics approval and consent to participate

Not applicable, under the data agreement of ADNI.

## Supplementary information

Interactive Plots of Clusters and Diagnosis Groups.

## Data Availability

The datasets generated during and/or analyzed during the current study are available in the ALZHEIMER’S DISEASE NEUROIMAGING INITIATIVE repository, http://adni.loni.usc.edu/.
